# Brain distortion and cerebrospinal fluid shifts in spontaneous intracranial hypotension

**DOI:** 10.1186/s12987-026-00878-3

**Published:** 2026-09-23

**Authors:** Amir El Rahal, Jürgen Beck, Katharina Wolf, Florian Volz, Manou Overstijns, Niklas Lützen, Charlotte Zander, Horst Urbach, Pierre Scheffler

**Affiliations:** 1 Department of Neurosurgery, Medical Center – University of Freiburg, Breisacher-Str. 64, 79106 Freiburg im Breisgau, Germany; 2 German CSF Center, Medical Center – University of Freiburg, Freiburg, Germany; 3 Department of Diagnostic and Interventional Neuroradiology, Medical Center – University of Freiburg, Freiburg, Germany; 4 Department of Diagnostic and Interventional Radiology, Medical Center – University of Freiburg, Freiburg, Germany

**Keywords:** Spontaneous intracranial hypotension, SIH, Brain sagging, Brain shift, AI, Convolutional Neural Network, Surgical closure, CSF Leak, CSF-venous fistula, Neurosurgery

## Abstract

**Background:**

Spinal cerebrospinal fluid (CSF) leaks in spontaneous intracranial hypotension (SIH) are assumed to cause intracranial CSF volume changes and brain sagging. The spatial pattern of CSF and brain volume changes, or shifts, remains incompletely characterized. We aimed to quantify CSF and brain shifts before and after surgical closure of spinal CSF leaks.

**Methods:**

We retrospectively analyzed 96 patients with SIH before and after surgical closure of their spinal CSF leaks. Artificial intelligence (AI)-assisted automated diffeomorphic registration and brain region segmentation of pre- and postoperative MRI imaging were used to extract a perioperative displacement map of the brain and the mean 3D displacement vector for each anatomical region. Regions were tested for significant changes using Hotelling’s T^2^ and compared against a healthy control cohort (*n* = 61) processed identically.

**Results:**

When comparing pre- and postoperative scans, brain structures demonstrated a consistent, predominantly upward-outward shift. Most major brain regions exhibited small, but statistically significant movement, with the highest magnitude seen in the parietal lobes and the left lateral ventricle (> 0.7 mm, *p* < 0.0001). Functionally connected regions, such as those within the fronto-limbic and visual networks, exhibited shifts in various directions, resulting in regional deformation. Displacement exceeded that of healthy controls roughly threefold (0.51 versus 0.16 mm across regions).

**Conclusion:**

Surgical CSF-leak closure in SIH leads to postoperative brain shift and tectonic-like deformation quantifiable on MRI. AI-assisted measurements reveal subtle brain movements and distortions, which could contribute to understanding of spinal leak pathophysiology and complement clinical assessment during follow-up.

**Supplementary Information:**

The online version contains supplementary material available at https://doi.org/10.1186/s12987-026-00878-3.

## Background

Spontaneous intracranial hypotension (SIH) is estimated to affect approximately 4 per 100,000 individuals annually. It most commonly presents with orthostatic headache and is frequently misdiagnosed [1–4]. Clinical presentation ranges from orthostatic headaches and audiovisual disturbances to severe cognitive impairment mimicking frontotemporal dementia. It results from a cerebrospinal fluid (CSF) leak or a CSF venous fistula along the spine [5–10]. These are classified as ventral dural tears (type 1), lateral or nerve-root-sleeve tears (type 2), and CSF–venous fistulas (type 3) [5].

Spinal CSF leaks can be associated with characteristic qualitative brain MRI findings, some of which have been aggregated into an SIH-specific scoring system, including reduced CSF cistern size, venous engorgement, pachymeningeal enhancement, and subdural effusions [11, 12]. These qualitative signs, assessed with the naked eye, constitute the hallmark of radiological assessment and are referred to as “sagging”. It implies a uniform sinking of the brain, which may oversimplify the three-dimensional displacement of intracranial architecture that occurs and cannot describe subtle changes or shifts in opposite directions that cancel out unless analyzed in more detail. We currently lack an understanding of how specific brain regions move relative to one another during the active disease state and, crucially, how they structurally reorganize.

The restoration of normal CSF dynamics following surgical spinal CSF leak closure offers a unique model to study this structural restitution [13–16]. However, quantifying these subtle shifts across the entire brain is challenging with conventional imaging techniques. Recent advances in artificial intelligence (AI) and automated diffeomorphic registration now enable precise voxel-wise mapping and displacement analysis between longitudinal scans [17–20]. 

In this study, we apply these AI-assisted tools to the pre- and postoperative MRI imaging of SIH patients to precisely quantify subtle global and regional perioperative brain shifts.

## Methods

### Ethics approval

The local ethics committee approved our study protocol (IDs: 22-1512-S1-retro/ 24-1296-S1-retro). The study was conducted in accordance with good clinical practice.

### Participants

In this single-center study from a tertiary referral center, we included patients with SIH who underwent surgery for a spinal CSF leak between April 2018 and December 2024 at our center and who had adequate pre- and postoperative MRI imaging. MRI imaging was considered adequate if it contained contrast-enhanced T1 isotropic images with a resolution of at least 1 mm. Preoperative imaging had to be performed within 12 months of surgery; postoperative imaging had to be conducted between 1 and 12 months after surgery. If multiple MRI scans were performed, the most recent preoperative scan and the postoperative scan that was closest to the 3-month postoperative follow-up were selected for our study. MRI images were also screened to exclude those showing subdural effusions or significant metal artifacts.

We compared the scans to the Beijing Normal University 2 (BNU 2) dataset from the Consortium for Reliability and Reproducibility (CoRR) [21]. This publicly available dataset includes 61 healthy young adults between 19 and 23 years old who were scanned twice with no intervention over an interval comparable to our follow-up (103–189 days between scans). The images were processed identically to the ones from the SIH group.

### Image processing

**Fig. 1:**
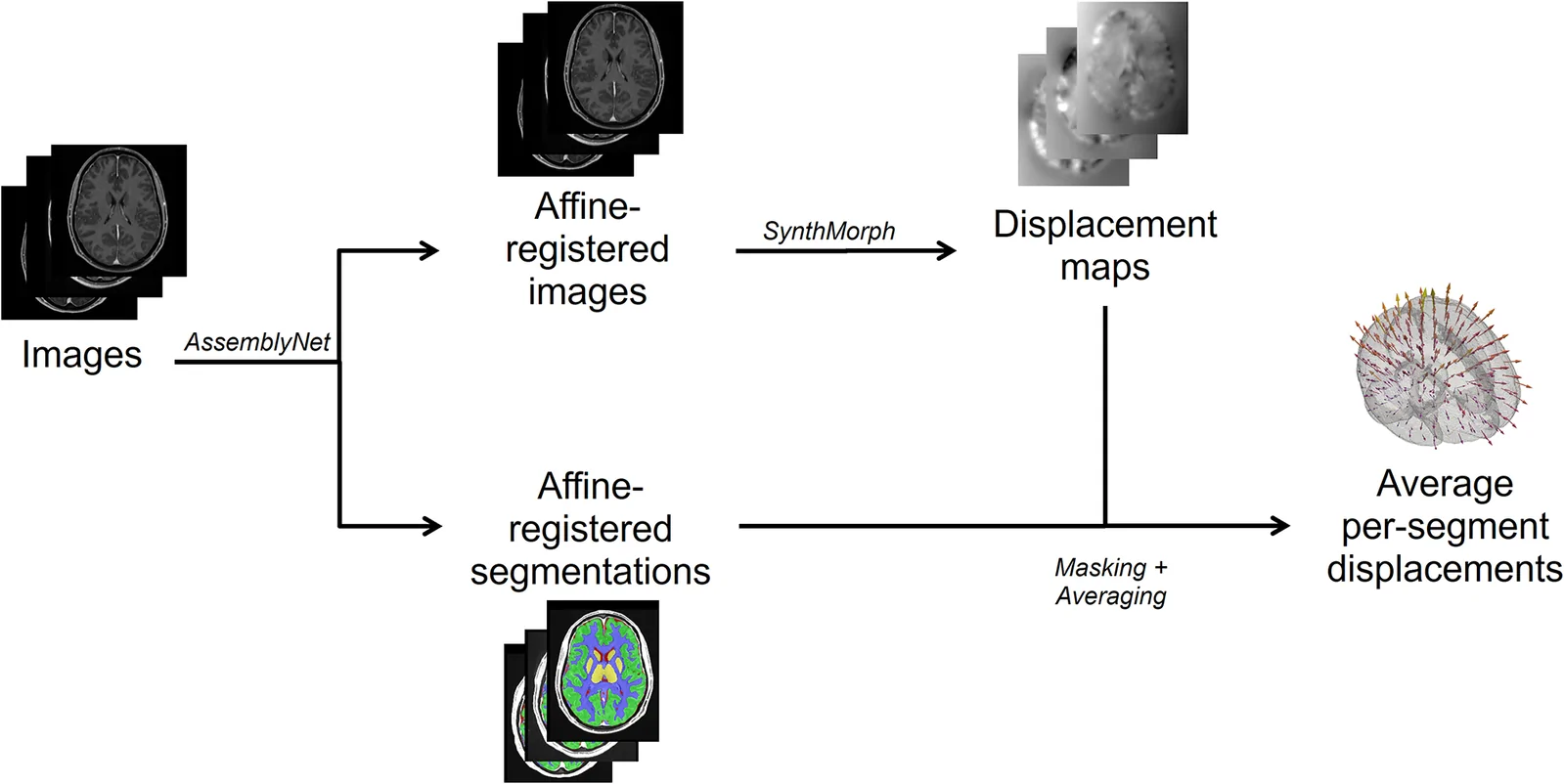
Schematic illustration of the processing pipeline. After conversion to NIfTI, images are affine-registered to an atlas image and then segmented into brain regions and structures using AssemblyNet. Then, SynthMorph performs diffeomorphic registration of pre- and postoperative pairs of affine-registered images to compute the image displacement maps. The segmentations from AssemblyNet are then used to isolate the displacements of specific brain regions

MRI images were anonymized and converted from DICOM to the NIfTI imaging format for further processing. The processing pipeline is illustrated in Fig. 1. Automated image segmentation for CSF spaces and the brain were then performed using the AssemblyNet model, which associates every voxel within the image with one or more out of 155 lobe, gyrus, or CSF space labels, as described by the Desikan-Killiany-Tourville protocol [20, 22]. All segmentations were visually verified by two authors (PS and AER). Diffeomorphic registration of pre- and postoperative images was performed using SynthMorph; this process associates every voxel within the preoperative image with the structurally corresponding voxel in the postoperative image, thereby allowing us to determine the three-dimensional displacement vector for each voxel [18]. Based on the segmented regions, we calculated the average displacement vector for each brain region. To test whether the findings depend on the registration algorithm, all pre- and postoperative pairs were registered a second time with ANTs SyN [23]. The affine-registered inputs and the segmentation were identical, and no parameter was tuned toward agreement, so the deformable algorithm was the only variable.

### Statistics

Statistical analysis was performed in Python. The distribution of brain region shifts across patients was tested for significant non-zero shifts using a one-sample Hotelling T^2^ test on the three-dimensional displacement vectors. Displacement was compared between leak types and between patients and controls using a two-sample Hotelling T^2^ test. The association between displacement and the interval from surgery to postoperative imaging was assessed with Spearman correlation, and by repeating regional analysis in patients imaged within a narrower 2–4 month follow-up window. Agreement between the two registration algorithms was quantified with Lin’s concordance correlation coefficient [24]. Confidence intervals (CI) are reported for displacement magnitude and for each directional component. Results are reported with mean and standard deviation or median and interquartile range (IQR) wherever applicable. P-values below 0.05 were considered significant; adjustment for multiple testing across the 155 regions was performed with the false discovery rate (FDR) using the Benjamini–Hochberg procedure.

## Results

### Patient characteristics

A total of 96 patients with adequate MRIs were included in the volumetric and displacement analysis as described in Table 1. Ventral type 1 leaks accounted for 53% of cases, lateral type 2 leaks for 32%, and type 3 CSF-venous fistulas for 14%. All the leaks were located in the thoracic region. The female-to-male ratio was approximately 5:3. The mean age was 43 ± 12 years. The preoperative MRI preceded surgery by a median of 6 days (IQR 4–16), the postoperative MRI followed surgery by a median of 98 days (IQR 78–123), and the two examinations were a median of 112 days apart (IQR 94–147).

**Table 1:** Characteristics of the patient population

***N*** ** (Patients)**	96
**Sex, ** ***N*** ** (%)**	
Female	60 (62.5%)
Male	36 (37.5%)
**Age at time of surgery, y**	
Mean ± SD	43.4 ± 11.5
**Type of Spinal CSF Leak, %**	
Type 1 leaks Type 2 leaks Type 3 leaks	53.1% 32.3% 14.6%

### Global pattern of brain shift

**Fig. 2:**
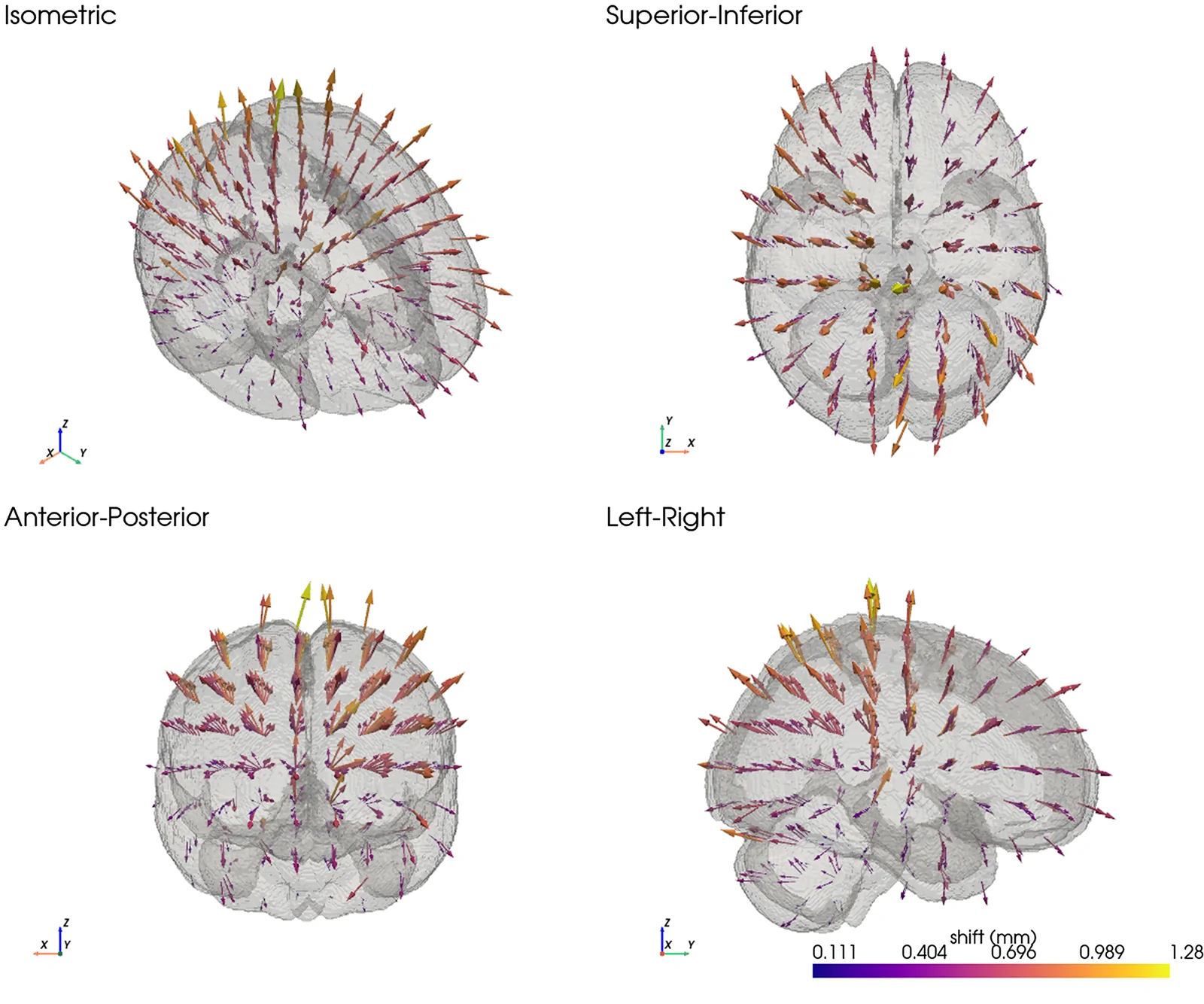
Illustration of the average brain shift from pre- to postoperative imaging from different perspectives. The mode of all included patient brains is represented as a grey, transparent wire mesh, overlaid with a quiver plot showing the direction and magnitude of the shift in the underlying region. The arrows are size- and color-coded based on the magnitude of the shift

Results of brain displacement are summarized in Fig. 2. This revealed a consistent global repositioning of the brain following surgical closure of the CSF Leak. The displacement field was characterized by a predominantly upward (superior) and outward shift of brain parenchyma. The average absolute magnitude of displacement was less than 1 mm (median 0.51 mm, IQR 0.38–0.66), with maximal mean shifts approaching 1.1 mm in individual regions.

### Shift of specific brain regions

Shifts for all major brain regions (all lobes and ventricles, the left and right cerebellar hemispheres, and the brainstem) are illustrated in Table 2. Across the full regional parcellation and after correction for multiple comparisons, 146 of 155 regions showed statistically significant displacement, indicating that the whole brain is being repositioned after leak closure.

**Table 2:** Average displacement of all major brain regions in 3 dimensions, sorted alphabetically. Magnitudes are given with a 95% confidence interval, and significance is reported against zero after FDR correction

Region	Left-Right	Posterior-Anterior	Inferior-Superior	Magnitude (95% CI)	*P*-value	Significant
3rd ventricle	-0.145	0.267	0.547	0.626 (0.42–0.88)	5.04*10^− 8^	Yes
4th ventricle	-0.083	-0.089	0.455	0.471 (0.23–0.78)	0.011	Yes
Brainstem	-0.059	0.015	0.343	0.348 (0.14–0.65)	0.027	Yes
Left cerebellum	-0.155	-0.191	0.118	0.273 (0.16–0.49)	5.91*10^− 4^	Yes
Left frontal lobe	-0.274	0.495	0.25	0.619 (0.45–0.82)	3.33*10^− 8^	Yes
Left lateral ventricle	-0.514	0.289	0.478	0.759 (0.59–0.98)	3.40*10^− 12^	Yes
Left occipital lobe	-0.147	-0.427	0.148	0.475 (0.35–0.64)	6.55*10^− 9^	Yes
Left parietal lobe	-0.279	-0.196	0.662	0.745 (0.51–1.02)	1.36*10^− 7^	Yes
Left temporal lobe	-0.212	0.115	-0.171	0.296 (0.16–0.47)	0.002	Yes
Right cerebellum	0.009	-0.396	0.07	0.403 (0.24–0.73)	4.80*10^− 5^	Yes
Right frontal lobe	0.124	0.321	0.204	0.400 (0.28–0.60)	2.37*10^− 5^	Yes
Right lateral ventricle	0.21	0.166	0.423	0.500 (0.34–0.76)	1.02*10^− 7^	Yes
Right occipital lobe	-0.141	-0.61	0.084	0.632 (0.46–0.87)	6.28*10^− 9^	Yes
Right parietal lobe	0.092	-0.415	0.662	0.787 (0.54–1.07)	1.69*10^− 7^	Yes
Right temporal lobe	0.078	-0.221	-0.239	0.335 (0.26–0.53)	4.73*10^− 7^	Yes

For major brain regions, the largest displacement magnitudes were observed in the parietal lobes (mostly upward) and the lateral ventricles (mostly upward and lateral shift), with mean shifts of up to 0.79 mm. Directionally, the displacement field was dominated by an upward component, present in approximately two-thirds of regions, with a weaker but consistent anterior component. Left–right displacement was more heterogeneous and region-dependent. The complete list of per-region displacements is provided in Supplement 1.

### Measurement floor and robustness

**Fig. 3:**
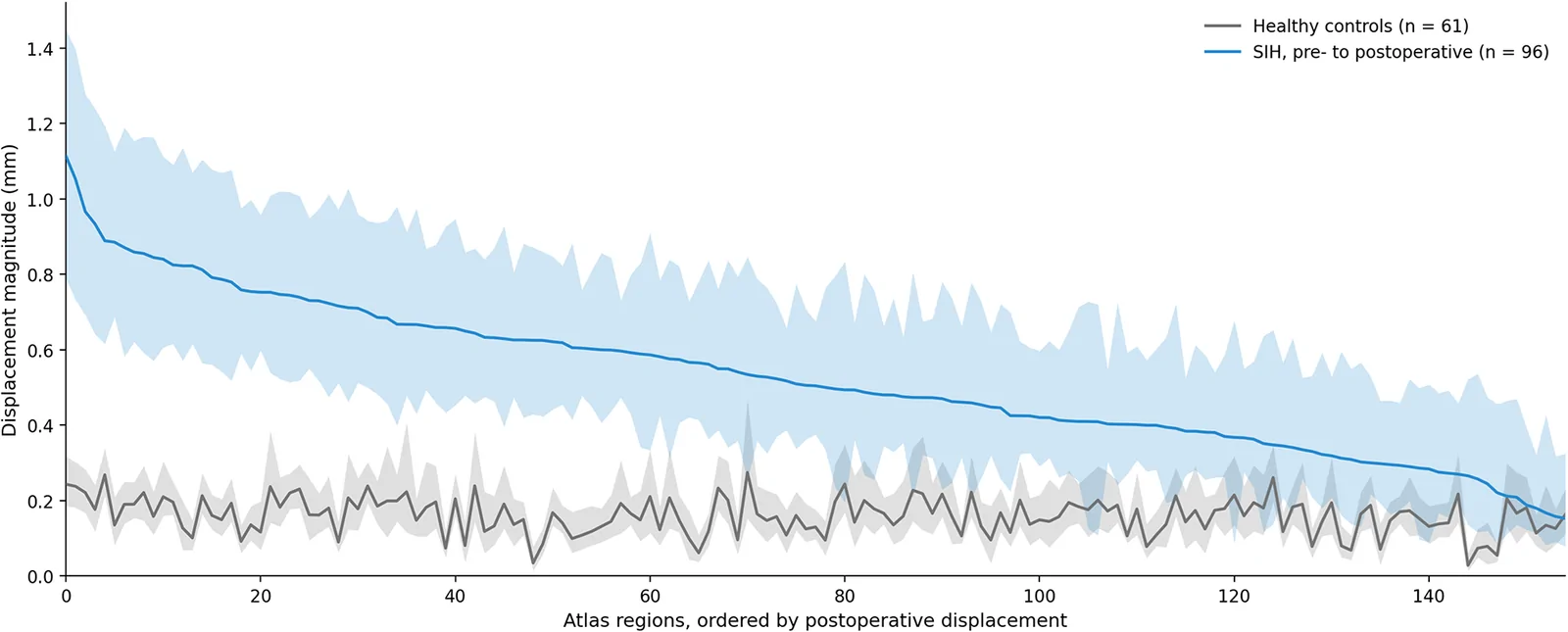
Mean displacement magnitude with 95% confidence intervals for each of the 155 atlas regions in patients with SIH between pre- and postoperative imaging (blue) and in healthy controls (gray). Regions are ordered by descending patient displacement; the control curve follows the same order

Processed identically, the displacement in the SIH cohort (median 0.51 mm across regions, IQR 0.38–0.66) was more than 3 times that of the healthy controls (median 0.16 mm, IQR 0.13–0.20), and 145 of 155 regions differed significantly from the healthy cohort (Fig. 3). Displacement did not depend on the timing of the postoperative scan: the median Spearman correlation with the surgery-to-imaging interval was − 0.08 and no region survived correction (Supplement 2). Restricting the analysis to a 2–4 month postoperative window (*n* = 58) left 137 significant regions, all of them also significant in the full cohort (Supplement 1).

**Fig. 4:**
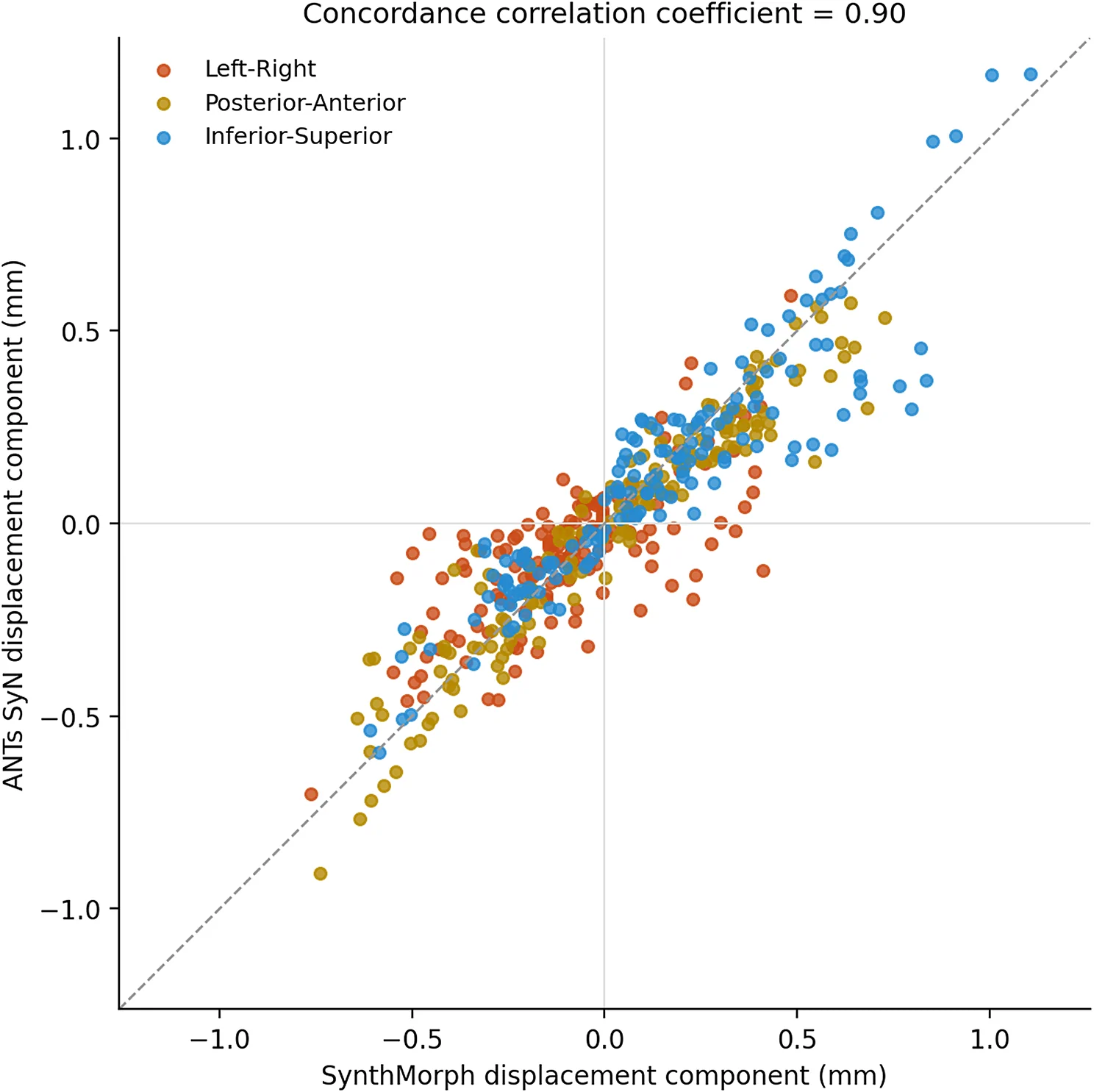
Mean regional displacement components from SynthMorph plotted against ANTs SyN for all 155 regions, giving 465 paired values across the left–right (red), posterior–anterior (yellow) and inferior–superior (blue) axes. Lin’s concordance correlation coefficient across all components was 0.90

Registering the same pairs with ANTs SyN reproduced both the direction and the size of the displacement with a concordance correlation coefficient of 0.90 across the three components of every regional vector (Fig. 4). ANTs recovered 125 of the 146 significant regions while adding two of its own (Supplement 1).

### Comparison of brain shift between leak types

**Fig. 5:**
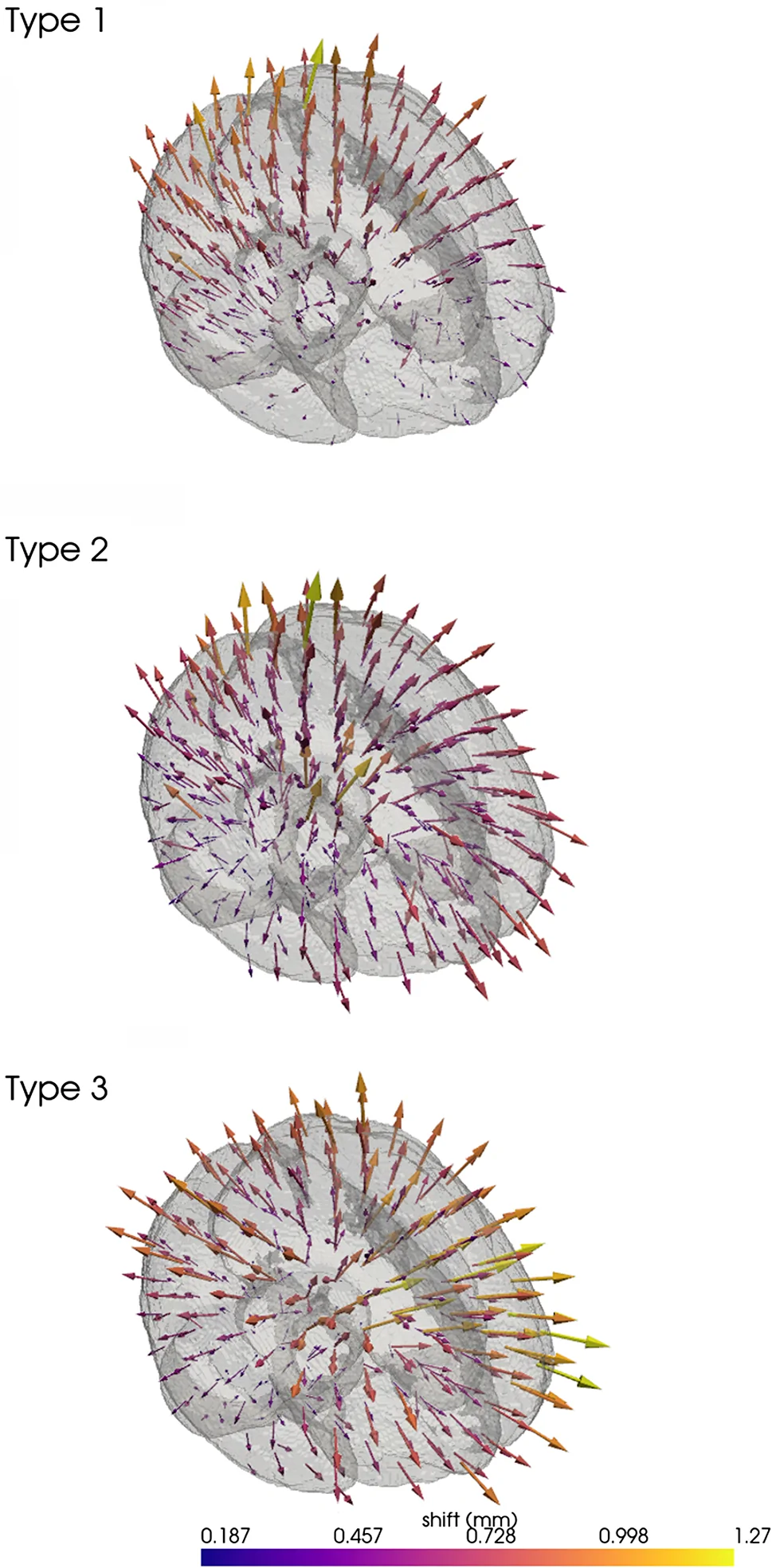
Comparison of the average brain shift from pre- to postoperative imaging between different leak types. The mode of all included patient brains is represented as a grey, transparent wire mesh, overlaid with a quiver plot showing the direction and magnitude of the shift in the underlying region. The arrows are size- and color-coded based on the magnitude of the shift

The displacement pattern stratified by leak type is shown in Fig. 5. No comparison between leak types survived FDR correction. Descriptively, the displacement fields differed, particularly when comparing Type 1 leaks with Types 2 and 3. Whereas Type 1 shows mostly an upwards shift in the parietal, central, and posterior frontal regions, Type 2 and 3 also show a relevant anterior shift of the anterior frontal lobes and an anterior-inferior shift of the temporal poles.

Table 3 provides an overview of the regions with the most significant differences in displacement; the complete statistical analysis of all areas is available in Supplement 3. Although some regions show statistical significance at the 0.05 level, after adjustment for multiple testing, no significant differences between leak types remain. Repeating the comparison on the independently registered ANTs fields did yield two significant regions for Type 1 versus Type 3 leaks, the brainstem and the fourth ventricle; these rank highest for that contrast in the primary analysis as well, so the result is suggestive but not robust to the choice of registration algorithm.

**Table 3:** Overview of brain region comparisons between leak types that reach the 0.05 significance level irrespective of multiple testing

**Type 1 vs. Type 2**		
**Region**	**Mean Diff. Magnitude (95% CI)**	***P*** **-value**
Lobules VIII-X	0.549 (0.27–1.11)	0.024
4th ventricle	0.486 (0.24–1.08)	0.028
Left cerebellum exterior	0.411 (0.22–0.82)	0.030
Left cerebellum	0.392 (0.20–0.82)	0.041
**Type 1 vs. Type 3**		
**Region**	**Mean Diff. Magnitude (95% CI)**	**P-value**
4th ventricle	0.590 (0.33–1.12)	0.001
Brainstem	0.469 (0.27–1.02)	0.001
Left thalamus	0.720 (0.38–1.29)	0.007
Cerebellum WM	0.390 (0.23–0.91)	0.008
3rd ventricle	0.516 (0.27–1.05)	0.010
Right cerebellum White Matter	0.529 (0.30–1.12)	0.010
Left temporal pole	0.550 (0.32–1.19)	0.012
Right thalamus	0.636 (0.34–1.17)	0.016
Right cerebellum	0.473 (0.26–1.12)	0.018
Cerebellum GM	0.356 (0.20–0.89)	0.018
Right cerebellum exterior	0.462 (0.25–1.12)	0.023
Left inf. lateral ventricle	0.448 (0.27–0.95)	0.024
Left anterior orbital gyrus	0.840 (0.39–1.69)	0.024
Right subcallosal area	0.392 (0.24–1.20)	0.028
Left lateral orbital gyrus	0.889 (0.39–1.73)	0.028
Lobules I-V	0.546 (0.26–1.06)	0.028
Right ventral DC	0.412 (0.21–0.97)	0.029
Left ventral DC	0.516 (0.25–1.05)	0.032
Subcortical GM	0.523 (0.25–1.01)	0.034
Left medial orbital gyrus	0.555 (0.27–1.33)	0.040
Right postcentral gyrus medial segment	0.609 (0.34–1.23)	0.042
**Type 2 vs. Type 3**		
**Region**	**Mean Diff. Magnitude (95% CI)**	**P-value**
Lobules I-V	0.608 (0.29–1.07)	0.040

Differences in magnitudes are given with a 95% CI. No comparison remained significant after correction for multiple testing across the 155 regions

### Shift of the ventricular system

**Fig. 6:**
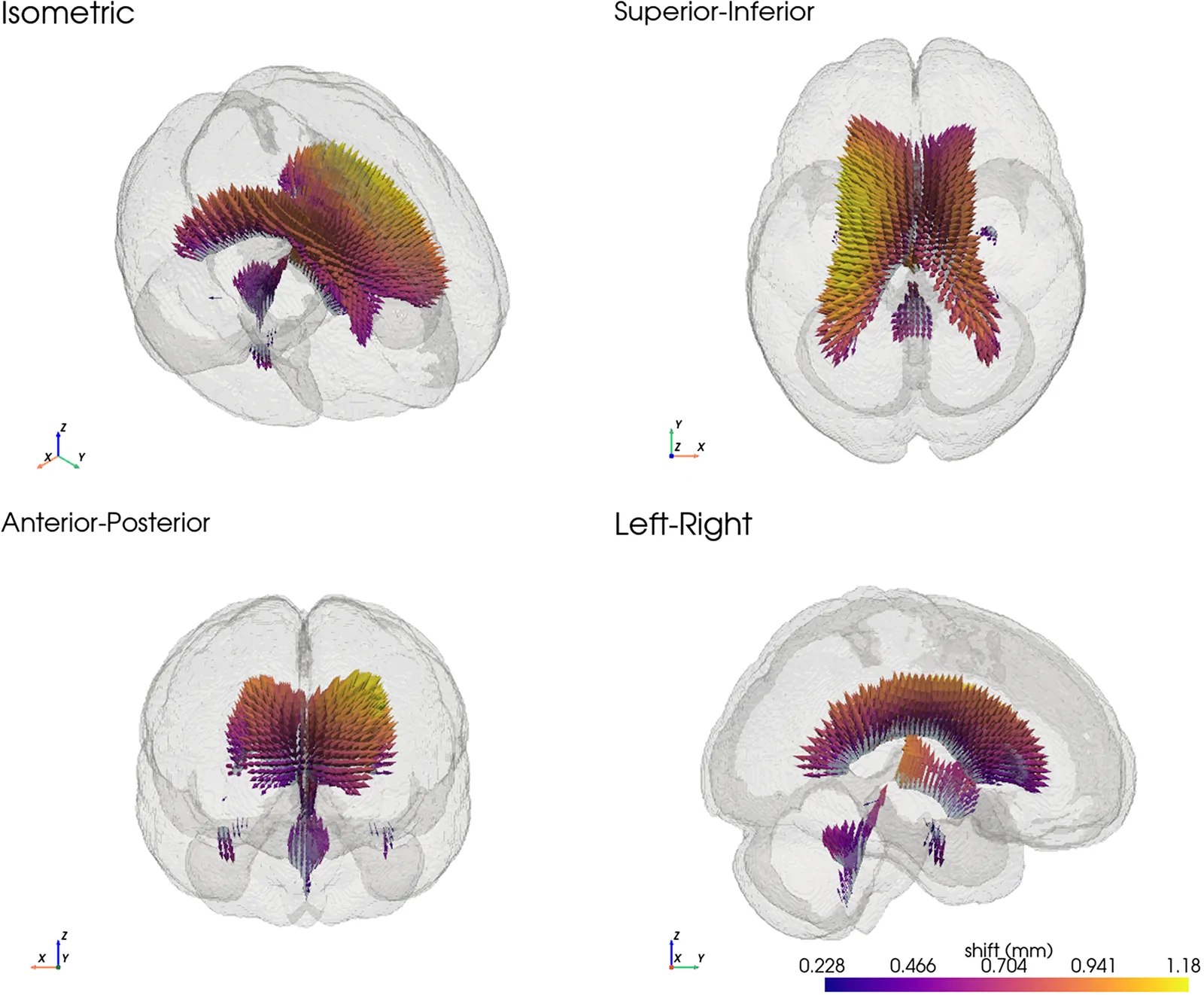
Illustration of the average ventricular shift from pre- to postoperative imaging from different perspectives. The mode of all included patient brains is represented as a grey, transparent wire mesh, overlaid with a quiver plot showing the direction and magnitude of the shift in the underlying region. The arrows are size- and color-coded based on the magnitude of the shift

Surgical closure of spinal CSF leaks was associated with a significant superior displacement of the ventricular system (Fig. 6). The lateral and third ventricles showed pronounced superiorly directed displacement (lateral ventricles up to 0.76 mm, *p* < 10⁻¹¹; third ventricle 0.62 mm, *p* < 10⁻⁷), whereas the fourth ventricle exhibited a smaller, directionally consistent upward shift. The temporal horn (right and left) showed a superior to inferior displacement.

### Displacement of fronto–limbic cognitive control networks

**Fig. 7:**
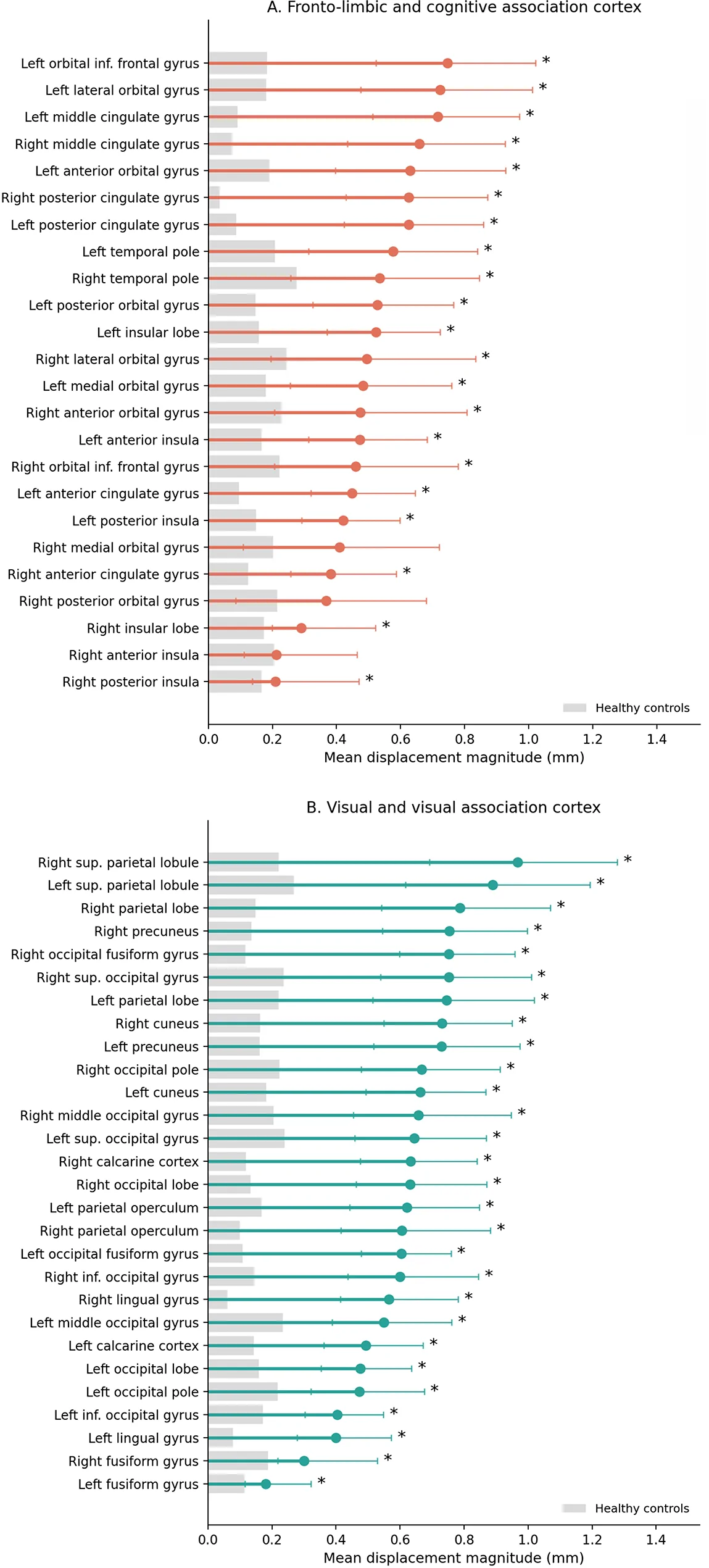
Paired lollipop plots showing mean displacement magnitude (mm) with confidence intervals; the mean magnitude in healthy controls is shown in gray. Panel **A** (red) shows the displacement of fronto-limbic and cognitive association cortices (orbitofrontal cortex, cingulate cortex, insula, temporal poles). Panel **B** (green) shows displacement of visual and visual association cortices, including the primary and secondary visual cortices (V1–V4) and regions along the dorsal and ventral visual streams. Asterisks indicate statistically significant displacement after correction for multiple testing

**Fig. 8:**
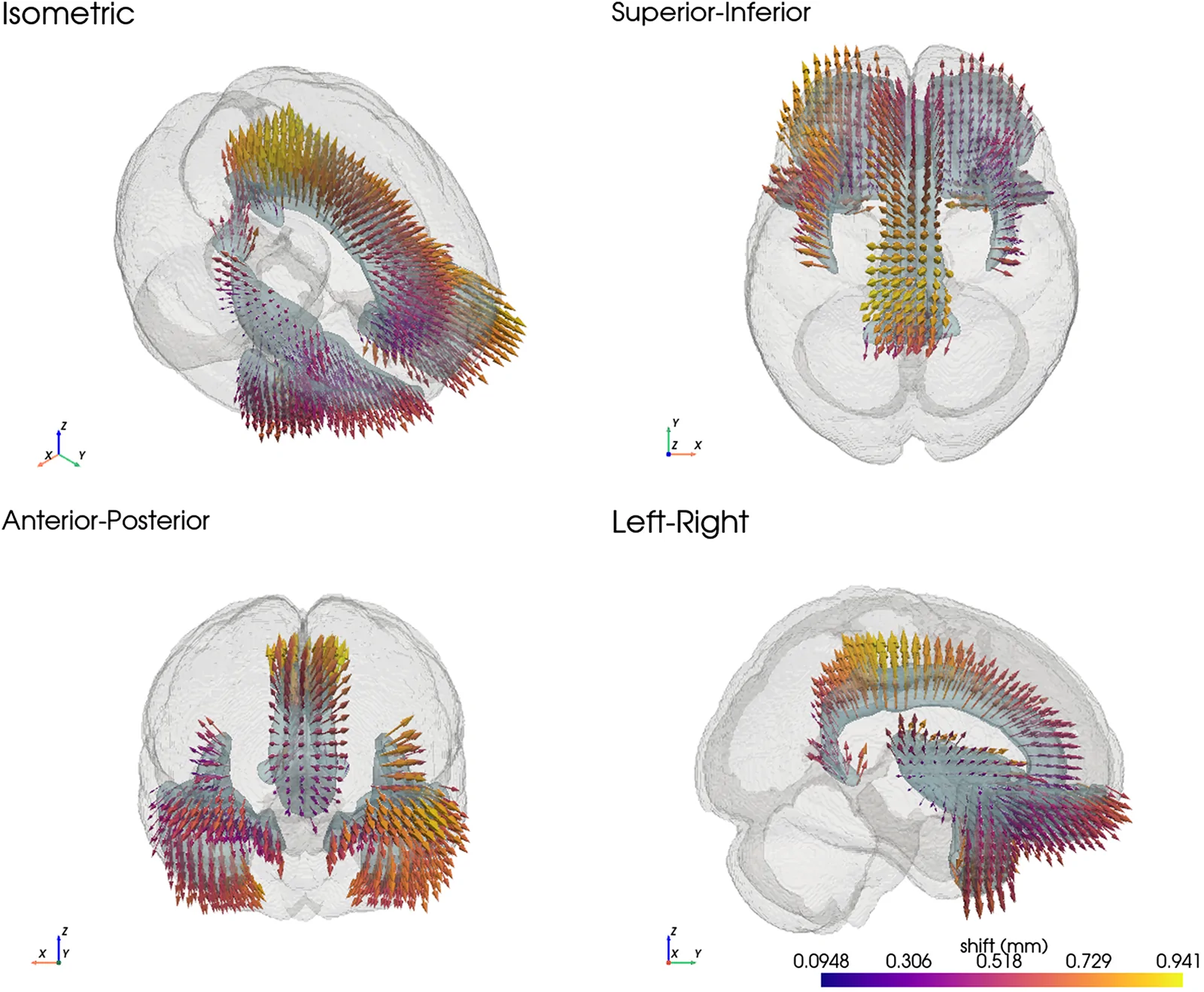
Illustration of the average displacement of fronto-limbic and cognitive association cortices (orbitofrontal cortex, cingulate cortex, insula, temporal poles) from pre- to postoperative imaging from different perspectives. The mode of all included patient brains is represented as a grey, transparent wire mesh, overlaid with a quiver plot showing the direction and magnitude of the shift

Our results demonstrated a consistent displacement within cortical and paralimbic regions. Within the orbitofrontal cortex, several subregions showed significant postoperative shift, predominantly on the left side, including the inferior, lateral, anterior, and posterior orbital gyri. In contrast, homologous right-sided regions demonstrated similar directionality but smaller magnitudes (Figs. 7A and 8).

The insula exhibited significant bilateral displacement with a predominantly upward–outward directionality. Focal displacement was also observed in the anterior temporal lobes, with both temporal poles showing significant remodeling. Within the cingulate cortex, significant bilateral displacement was identified in the middle and posterior cingulate gyri. Together, these findings indicate a sub-millimeter, regionally specific postoperative displacement across frontal–limbic cognitive control networks.

### Displacement of visual and visual association networks

**Fig. 9:**
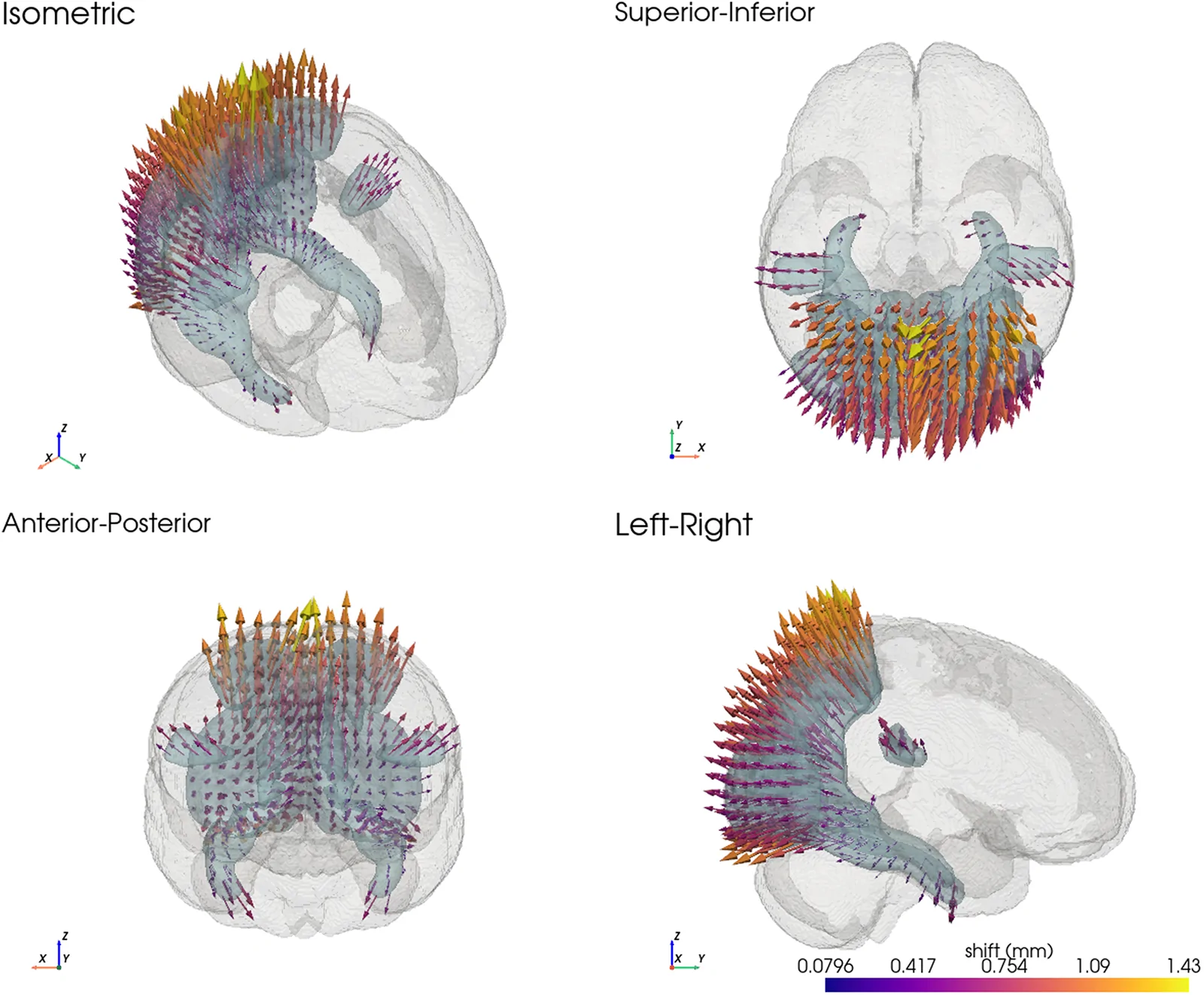
Illustration of the average displacement of visual and visual association cortices, including the primary and secondary visual cortices (V1–V4) and regions along the dorsal and ventral visual streams. from pre- to postoperative imaging from different perspectives. The mode of all included patient brains is represented as a grey, transparent wire mesh, overlaid with a quiver plot showing the direction and magnitude of the shift in the underlying region. The arrows are size- and color-coded based on the magnitude of the shift

Displacement analysis demonstrated significant involvement of the primary and secondary visual cortices (V1–V4) and higher-order visual association networks, with bilateral primary visual cortex (V1) displacement reaching statistical significance (*p* < 0.00001) (Figs. 7B and 9). Regions along the dorsal visual stream, including occipital and parietal cortices, showed consistent displacement patterns. In contrast, structures along the ventral visual stream, particularly inferior temporal areas, also exhibited significant postoperative shifts.

## Discussion

### Micro-distortions of the brain and inner brain shift – A “tectonic” view beyond brain sagging

Our results indicate that in SIH, spinal CSF leaks lead to specific brain shifts and parenchymal distortions that extend beyond the traditional concept of brain sagging. Using AI-assisted diffeomorphic registration and analysis, we demonstrate that these changes reflect region-specific whole-brain displacement [18]. Surgical closure of spinal CSF leak leads to a significant upward and outward shift of brain structures. These findings complement established brain MRI signs of SIH, including reduced basal cisterns, smaller ventricles, subdural hematoma, and distended cortical veins. Our key findings demonstrate that spinal leaks induce whole-brain deformations that can be quantified and may be longitudinally monitored (Fig. 2; Table 2, Supplement 1).

We propose the term “brain tectonics” to refer to the brain distortions that happen in spontaneous intracranial hypotension. Analogous to geological tectonics, where shifting continental plates create stress, deformation, and tremors at fault lines, the displacement of brain areas in different directions can exert mechanical tension on various structures. This framework may be part of the clinical heterogeneity as encountered in SIH. One might speculate that the clinical phenotype depends, at least in part, on which brain areas bear the brunt of the mechanical stress.

### Comparison of leak types

The visual representation of displacements across leak type suggests differences in brain shift. Closure of type 1 leaks is primarily associated with upward movement, particularly of the most apical brain regions, such as the central area, the parietal lobes, and the posterior frontal lobes (Fig. 5). In contrast, Type 2 and Type 3 leaks show a more complex displacement configuration, with additional anterior and anterior–inferior components, particularly involving the frontal base and temporal poles. However, despite these visually apparent differences, per-region statistical analysis did not reveal significant leak-type–specific displacement after correction for multiple testing (Table 3). The cohort as a whole is not small, but the subgroups are, and only large between-type differences could have been detected; our analysis may have been underpowered. Another explanation is that per-region analysis averages all displacements within a region, potentially overshadowing focal differences. For example, the frontal gyri span from the central region to the frontobasis; any differences in the movement of the anterior parts may be statistically imperceptible compared to the movement of the apical parts of the gyri. From a mechanistic perspective, differences in patterns among leak types could, for example, reflect discrepancies in the severity and continuity of CSF loss [25].

### Specific regions with significant shifts

The regional displacement pattern shows consistent involvement of the orbitofrontal cortex, the anterior and middle cingulate cortex, the insula, and the anterior temporal regions. These regions together form key nodes of the fronto–limbic and salience networks [26–29]. One can speculate that sub-millimeter displacements of these regions could potentially disrupt network integration rather than focal cortical function. Clinically, these subtle brain changes may underlie the executive dysfunction, attentional deficits, cognitive slowing, and “brain fog” frequently reported in SIH, even in the absence of structural lesions or atrophy [10]. The displacements after leak closure support a functional, dynamic mechanism that could help explain why SIH can mimic syndromes such as behavioral-variant frontotemporal dementia, and why these symptoms can improve rapidly after leak closure [6, 10, 30].

Tectonic displacement affecting the primary and secondary visual cortices (V1–V4), posterior cingulate, and parietal regions, which together support spatial and associative visual processing, are also significantly altered [31–33]. In addition, inferior–superior brain displacement may exert traction on biomechanically coupled pathways such as the optic nerves and chiasm, which are not directly segmented in our analysis. More generally, this global reconfiguration can disrupt, for example, visuospatial integration, leading to blurred vision and visual instability, which are commonly observed in SIH [34].

Significant displacement of the temporal poles, transverse temporal regions, insula, and adjacent opercular cortex could suggest involvement of the auditory network. These cortical shifts might contribute to hearing-related symptoms such as muffled sounds, tinnitus, and sound distortion, which are often bilateral and posture-dependent [35–37]. This cortical mechanism may complement the existing hypothesis regarding pressure changes within the inner ear [38, 39]. 

The mechanisms behind the subtle, region-specific perioperative repositioning remain speculative. Inspiration drives CSF upward along the spinal canal and contributes to filling the ventricular system against gravity, a contribution a chronic spinal leak would be expected to blunt [40]. The superior displacement of the ventricular system after leak closure is compatible with the restoration of this mechanism, although our data are anatomical rather than flow-based and cannot test it directly.

Collectively, our regional segmentation and displacement analysis introduce a biomechanical model of SIH, in which spinal CSF leaks induce mesoscale brain shifts and whole-brain deformation rather than isolated, one-directional “sagging” of specific structures. Although its clinical implications remain highly speculative, this tectonic framework may provide a new mechanistic insight into the pathophysiology of SIH. It may help to explain the characteristic constellation of cognitive, visual, auditory, and pain-related symptoms.

### Limitations of the study

Several limitations should be considered when interpreting these findings. First, the analysis relies on postoperative imaging changes to infer pathological brain shifts in patients with SIH. This interpretation assumes that preoperative brain position is pathological and that the intervention restores normal anatomy. While this assumption is well-supported by established SIH pathophysiology, a direct comparison with pre-disease baseline imaging is not feasible given the rarity of SIH and the absence of premorbid MRI.

In addition, the approach relies on AI-assisted diffeomorphic registration and segmentation of imaging data. Compared to manual registration and segmentation, AI-based methods are faster and more reproducible, but remain susceptible to algorithmic errors that may be difficult to characterize. Confirming our results with a second registration algorithm demonstrates the reliability of our method, but both registration methods share discretization and interpolation assumptions that may lead to systematic errors. Nevertheless, given the sub-millimeter effect size in some areas and the need for whole-brain, longitudinal analysis, AI-based methods currently represent the most viable and robust solution.

All images were acquired on MRI scanners within our department with a standardized protocol for SIH. Most images were acquired on a single device (Siemens Avanto 1.5T, Siemens Healthcare, Erlangen, Germany), but some pre- and postoperative examinations may have been performed on a different device, which can cause variability in the resulting displacement maps. The control cohort also differs from the patients in age and in the use of contrast; even though SynthMorph is designed to be contrast-invariant, this may reduce comparability between groups.

It remains unclear how fast the postoperative displacements we describe develop. Although displacement did not correlate with the surgery-to-imaging interval in our analysis, longitudinal imaging follow-up is necessary to evaluate the speed of recovery from SIH. Additional postoperative examinations exist for some of the included patients, but they were usually obtained for clinical reasons, often because of new or recurrent symptoms, and are of variable quality, so a time-course analysis in our population would be biased by the reason for re-imaging.

## Conclusions

Surgical closure of spinal CSF leaks in SIH is associated with significant upward and outward restitution of brain position and whole-brain tectonic deformation, with a pattern that can be identified using AI-assisted MRI analysis. Our framework establishes a more detailed and objective characterization of brain displacement and may provide explanations for the diverse clinical manifestations of SIH. Such displacement patterns may also serve as an additional imaging marker of disease state and postoperative recovery.

## Supplementary Information

Below is the link to the electronic supplementary material.

Supplementary Material 1

Supplementary Material 2

Supplementary Material 3

## Data Availability

Parts of the dataset containing data from patients who agreed to share it with other researchers will be made available upon reasonable request to the corresponding author.

## Abbreviations

AI: Artificial intelligence
CSF: Cerebrospinal fluid
FDR: False discovery rate
MRI: Magnetic resonance imaging
SIH: Spontaneous intracranial hypotension
